# Generation of Linear Traveling Waves in Piezoelectric Plates in Air and Liquid

**DOI:** 10.3390/mi10050283

**Published:** 2019-04-27

**Authors:** Alex Díaz-Molina, Víctor Ruiz-Díez, Jorge Hernando-García, Abdallah Ababneh, Helmut Seidel, José Luis Sánchez-Rojas

**Affiliations:** 1 Microsystems, Actuators and Sensors Group, Universidad de Castilla-La Mancha, E-13071 Ciudad Real, Spain; victor.ruiz@uclm.es (V.R.-D.); jorge.hernando@uclm.es (J.H.-G.); joseluis.saldavero@uclm.es (J.L.S.-R.); 2 Electronic Engineering Department, Hijjawi Faculty for Engineering Technology, Yarmouk University, 21163 Irbid, Jordan; a.ababneh@yu.edu.jo; 3 Chair of Micromechanics, Microfluidics/Microactuators, Faculty of Natural Sciences and Technology, Saarland University, 66123 Saarbrücken, Germany; seidel@lmm.uni-saarland.de

**Keywords:** traveling waves, piezoelectric, microactuator, MEMS

## Abstract

A micro- to milli-sized linear traveling wave (TW) actuator fabricated with microelectromechanical systems (MEMS) technology is demonstrated. The device is a silicon cantilever actuated by piezoelectric aluminum nitride. Specifically designed top electrodes allow the generation of TWs at different frequencies, in air and liquid, by combining two neighboring resonant modes. This approach was supported by analytical calculations, and different TWs were measured on the same plate by laser Doppler vibrometry. Numerical simulations were also carried out and compared with the measurements in air, validating the wave features. A standing wave ratio as low as 1.45 was achieved in air, with a phase velocity of 652 m/s and a peak horizontal velocity on the device surface of 124 μm/s for a driving signal of 1 V at 921.9 kHz. The results show the potential of this kind of actuator for locomotion applications in contact with surfaces or under immersion in liquid.

## 1. Introduction

Microelectromechanical systems (MEMS) are key components for the progress of miniaturization in disciplines such as consumer electronics, instrumentation, and healthcare [1]. Recently, robotic research on the micro and millimeter scales (i.e., the insect scale) has also benefited greatly from technological advances in MEMS and other enabling technologies, such as additive manufacturing, piezoelectric actuators, and low power sensors [2,3]. In this field, the principles of locomotion are diverse [4,5]. Wave-based locomotion can be adapted to miniature systems, as already happens in nature [6]. The type of wave can be either standing or traveling; however, traveling waves (TWs) are usually preferred, due to the absence of contact tips and the simplicity of bidirectional motion.

Circular motors based on TWs are already well established, with the actuation of two degenerate modes and a proper spatial shift [7]. Similarly, linear motors have also been accomplished by combining two modes at the same frequency, with either two bending modes [8] or a bending mode and a longitudinal mode [9,10]. Most of these approaches are millimeter-sized; however, their assembly is not commonly based on silicon monolithic technology, which would allow for cost reduction, manufacturing precision, and the possibility of system-level integration [11].

It is equally interesting to mention that linear motors can also be implemented by just combining two contiguous bending modes, without the degeneration requirement, which results in less restrictions on the design of the device [12]. Different reports have already demonstrated centimeter-sized devices that move on solid surfaces with this approach [13,14,15]. Another important field to consider is locomotion within liquids, which emulates the movement of aquatic animals [16]. An example is to mimic the structure of a cuttlefish or a water flatworm, with two moving membranes attached to the length of the body, which has already been considered for macro-scale systems [17,18,19].

Here we present millimeter-sized linear TW actuators based on piezoelectric aluminum nitride (AlN) on silicon plates, fabricated with MEMS technology. The structure is a cantilever, with the proper top electrode layout to realize progressive waves along its width, mimicking the animal membranes mentioned before. TWs were developed by combining two neighboring modes, and the feasibility of the approach was demonstrated for two different excitation frequencies on the same device, by coupling different pairs of modes. Both the simulations and experimental results were compared, and the investigations were realized in air and liquid. For the TWs in air, a standing wave ratio (SWR) as low as 1.45 was determined, with a phase velocity of 652 m/s and a peak horizontal velocity of 124 μm/s for a 1 V peak amplitude and a frequency near 900 kHz.

## 2. Materials and Methods

**Figure 1:**
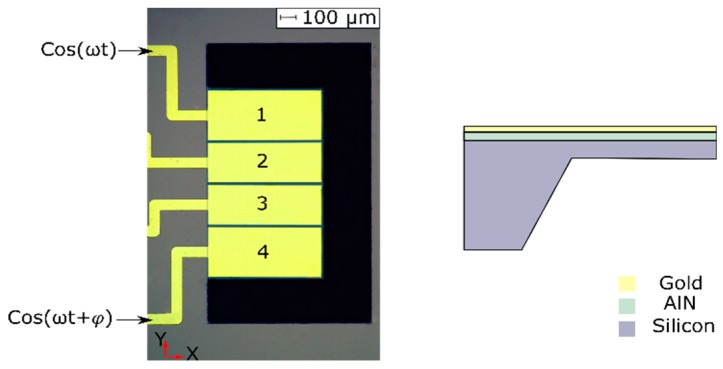
Optical micrograph of the 750 μm × 1300 μm cantilever and cross section view along the length of the device, including the suspended part and the silicon anchor.

The size of the cantilever under study is 750 μm × 1300 μm (Figure 1). It was fabricated out of a low resistivity silicon p-doped wafer of 520 μm thickness, which served as the bottom electrode. For the piezoelectric layer, 1 μm AlN was sputtered, with a measured *d*_33_ of 3.22 pm/V. The deposition was realized with a back-pressure level of 4 × 10^−^^3^ mbar and a sputtering ratio close to 20 nm/min at 1000 W. A sputtered gold layer was used for the top electrode with a thickness of 400 nm. A silicon membrane was obtained by wet etching with 38% potassium hydroxide (KOH) at 85 °C, previously depositing a 400 nm thick Si_3_N_4_ layer on the backside using plasma-enhanced chemical vapor deposition (PECVD). This allowed us to pattern the silicon substrate to a residual thickness of about 40 μm in the areas where the suspended beams were fabricated. Finally, the plates were released by a deep reactive-ion etching (DRIE) process. Additional details of the fabrication process can be found in [20].

The top electrode design comprises 4 isolated strips, which have been previously reported for the actuation of roof tile-shaped modes in liquid sensor applications [21]. Here, only the two outer electrodes (1 and 4 in Figure 1) were used for the generation of TWs.

The displacement of the device versus time and versus frequency was measured by means of a laser Doppler vibrometer (Polytec MSV 400, POLYTEC, Waldbronn, Germany). This instrument allows for the measurement of the out-of-plane displacement through a laser spot, which scans a set of points distributed along the surface of the cantilever.

Finite elements method (FEM) software was used to corroborate the generation of the TWs. The software ‘Coventorware’ was employed, and a 3D model of the cantilever was analyzed with the help of modal harmonic analysis. In the simulated design, the angle of inclination of the substrate due to the wet etching process (shown in Figure 1) was taken into account, as well as the surrounding bulk material, where the clamped boundary condition was applied. The mesh used was composed of 9255 3D cubic parabolic elements. The dimensions of the device were the nominal values, and the elastic and rest of the material constants were as provided by the software, except for the piezoelectric constant of AlN, which was coincident with the measured value. A good agreement between the measured and calculated resonance frequencies was attained, without including any built-in stress in the structure. This was confirmed with measurements of the plate deformation by an optical profiler, obtaining values close to 773 and 171 nm along the lines perpendicular and parallel to the anchored side of the plate, respectively.

Before the investigation of the TW generation, the static piezoelectric response was measured with the laser Doppler vibrometer, applying an 8 V peak amplitude to the top four electrodes at a frequency of 200 Hz (far from any neighbor resonance). The deflection reached a maximum value of 3.1 nm/V, located at the edge of the tip.

## 3. Results and Discussion

**Figure 2:**
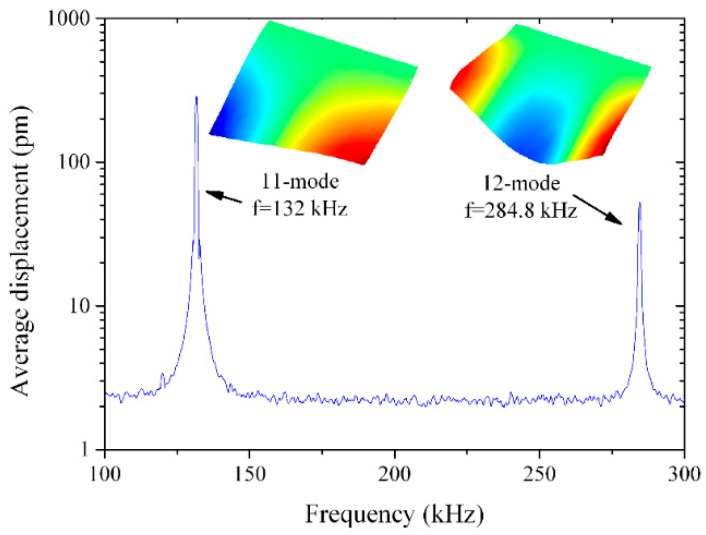
Measured frequency response of the cantilever from 100 to 300 kHz by means of a laser Doppler vibrometer. Modes (11) and (12) were detected. The measured modal shapes are also included.

First, we present the frequency response of the cantilever obtained by means of the laser Doppler vibrometer, by applying a periodic chirp signal to Electrode 1. Figure 2 shows the average displacement versus the frequency from 100 to 300 kHz. Two modes were detected, Modes (11) and (12), following Leissa nomenclature [22]. Mode (11) was antisymmetric, while Mode (12) was symmetric, with respect to the center of the cantilever.

By applying a sine wave signal to each of the outer electrodes, with a phase difference, φ, and a frequency, $\omega_{f},$ between the two modes previously mentioned, the displacement of the device, in the two-mode approximation, can be described by

(1) $$w\left(x,y,t\right)=\left[Q_{s}\Phi_{s}\left(x,y\right)+Q_{a}\Phi_{a}\left(x,y\right)\right]\cos\left(\omega_{f}t\right)+\left[Q_{s}\Phi_{s}\left(x,y\right)-Q_{a}\Phi_{a}\left(x,y\right)\right]cos(\omega_{f}t+\varphi ),$$

which is the superposition of the displacements associated with the respective sine waves, applied to each of the outer electrodes. $\Phi_{s}$ and $\Phi_{a}$ represent the shapes of the symmetric and antisymmetric modes, respectively and $Q_{s}$ and $Q_{a}$ are the weights determining the contribution of each of these modes. As is shown in [23], this equation can be deconstructed into four different progressive wave terms, and, depending on the values of $Q_{a}$, $Q_{s}$, and φ, the sum of these terms results in either a traveling or standing wave.

**Figure 3:**
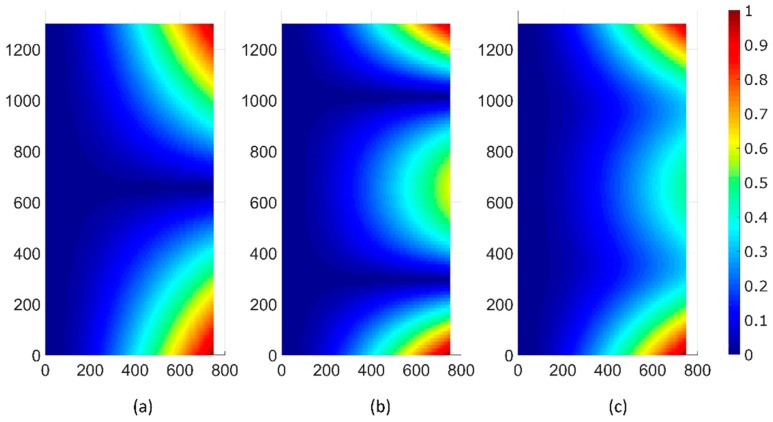
Envelope of the maximum displacement over the cantilever surface of (**a**) Mode (11), (**b**) Mode (12), and (**c**) Equation (1) with the parameters mentioned in the text. The color scale represents the unitary displacement.

This idea is depicted in Figure 3 where we compare the envelope of the maximum displacement of the modes in Figure 2 and that of a progressive wave resulting from the combination of these modes, as in Equation (1). Figure 3a,b correspond to Modes (11) and (12), respectively. Figure 3c is the envelope given by Equation (1), for $Q_{a}=1.3$, $Q_{s}$ = 1, $\omega_{f}=1.31\times 10^{6}\;\mathrm{rad}/s$, and φ=90°. Although these values were chosen only for illustration purposes, while the modes (standing waves) were characterized by nodes along the width of the cantilever edge (dark blue in the figure), the displacement associated with the combination of modes in quadrature resulted in a profile without nodes along the edge, which is characteristic of a progressive wave.

**Figure 4:**
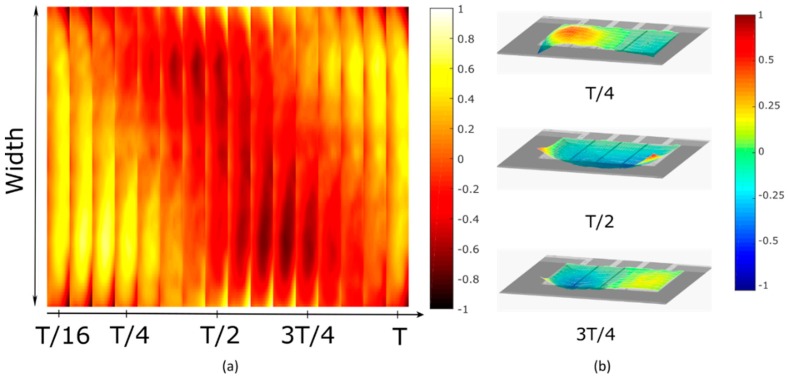
Time frame of the measured traveling wave (TW) by combining Modes (11) and (12): (**a**) 2D representation and (**b**) 3D representation (a video clip is available (Video S1)). The color scale represents the normalized displacement of the cantilever. T corresponds to the period of the TW.

This basic analytical calculation suggests that a TW may be generated just by using electrodes that excite the two modes with different weights ($Q_{s}$ ≠ $Q_{a}$). Next, we present the experimental measurements on the device described above, whose electrodes can be shown to satisfy this condition [24]. The following parameters were chosen for the sine waves applied to the two outer electrodes: a frequency of 208.4 kHz, corresponding to the average of the resonant frequencies of the two modes, a 1 V peak amplitude, and a phase shift, φ of 90°. Measurements were taken in the time domain with the laser Doppler vibrometer. Figure 4a shows the map of the displacement on the device surface (anchored on the left side) at different instants. The plot shows clearly that the peaks and valleys are located in different places depending on the moment in time as the wave travels along the width. This differs from a standing wave-based modal response where the maxima and the minima of the wave maintain their location independently of the instant in time. Figure 4b shows different snapshots of the 3D TW as it propagates from right to left. An animation of the 3D movement can be seen in Video S1. A TW in the opposite direction can be obtained by simply exchanging the sine waves applied to the outer electrodes. The TW envelope reaches a maximum value of 37 pm, and the phase velocity can be estimated as 416 m/s. The horizontal velocity of a point on the surface of the cantilever was estimated to be 3 μm/s for the voltage applied [25]. A pure TW would have a SWR of 1. In our case, due to the free-free boundary condition at the edges, the wave was not ideal. Excluding the edges of the plate, the SWR reached a value of 1.46 for a centered window covering 54% of the total width of the plate. This value is comparable to those reported for centimeter-sized devices [26,27].

**Figure 5:**
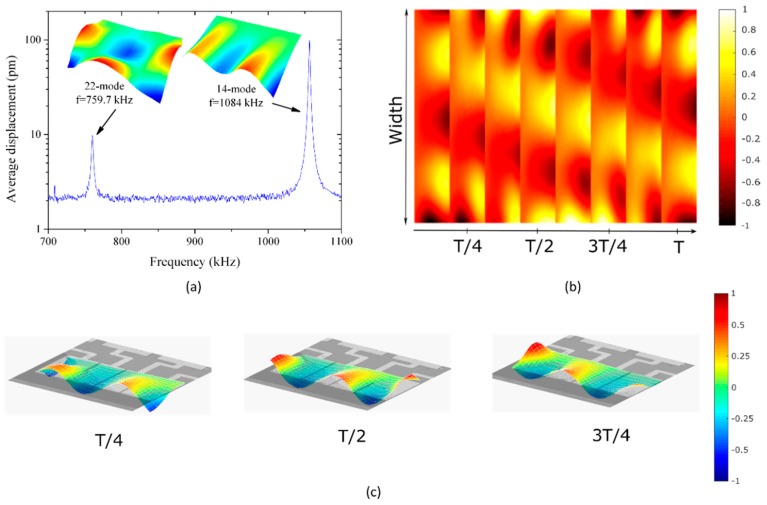
(**a**) Measured frequency response of the cantilever from 700 kHz to 1.1 MHz. Time frame of the measured TW by combining Modes (22) and (14). (**b**) 2D representation and (**c**) 3D representation (a video clip is available (Video S2)). The colors represent the normalized displacement of the cantilever. T corresponds to the period of the TW.

TWs may be generated by combining different couples of modes on the same device. Figure 5a shows the frequency response of the cantilever in a different range between 700 kHz and 1.1 MHz. In this case, the symmetric mode corresponded to Mode (22) and the antisymmetric one to Mode (14). By applying the same excitation as for the previous case but adapting the frequency to the new average (921.9 kHz), we obtained the response of Figure 5b, again characteristic of a TW motion, reaching 122 pm of maximum vertical displacement. The estimated phase velocity was 652 m/s, and the horizontal speed on the surface was 124 μm/s. The SWR reached a value of 1.45 in a central window corresponding to 93% of the total width of the plate. A 3D representation of the TW at different instants in time is shown in Figure 5c. Video S2 shows the 3D movement of this TW.

To gain a more complete understanding of the generation of a TW and the effect of the different parameters on both the device and the excitation signals, the experimental results were compared to a FEM model. The FEM analysis provides a more realistic and quantitative description of the device behavior as compared to the basic analytical approach given by Equation (1), as it allows the inclusion of the real shape of the surrounding anchor (see the cross section of Figure 1), the electromechanical coupling for the given geometry of the electrodes, and the contribution of those modes further away from the two modes considered to determine the frequency of the actuation. 

**Figure 6:**
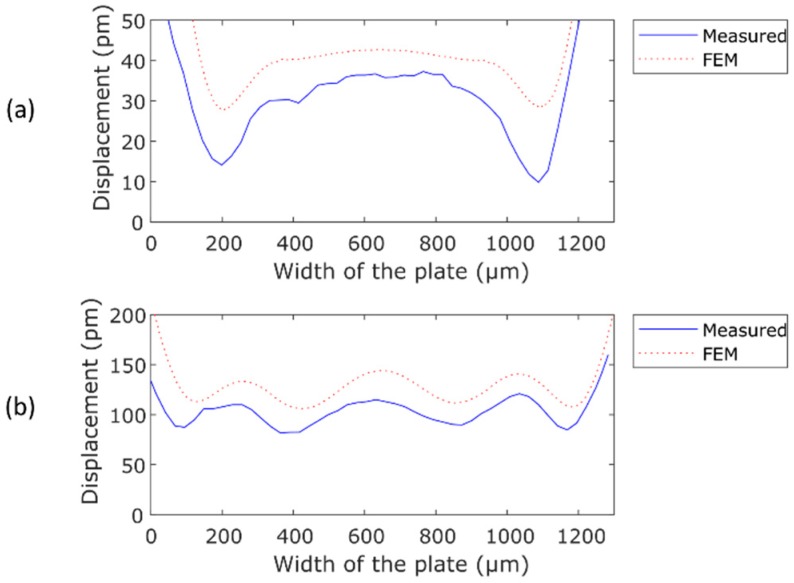
Comparison between the envelope of the measured (blue) and the FEM simulated (red) TW: (**a**) combination of Modes (11) and (12) at 208.4 kHz and (**b**) combination of Modes (22) and (14) at 921.9 kHz.

Figure 6a,b show a comparison between the measured and the simulated envelopes of the maximum displacement along the width of the plate, close to the edge of the cantilever, for the two cases presented above. It can be seen that there is a reasonable agreement between the experiments and the modeling, which supports our approach. The discrepancy observed may be related to the slight differences between the calculated frequencies and modal shapes and those in the real structure, induced by a non-ideal anchoring. This hypothesis has been checked by the FEM model—varying the portion of the substrate, that is included in the calculation, changed the modal frequencies, affecting the resulting TW envelope. We found that the TW envelope, in shape and amplitude, was very sensitive to the shape and frequency of the neighbor modes.

**Figure 7:**
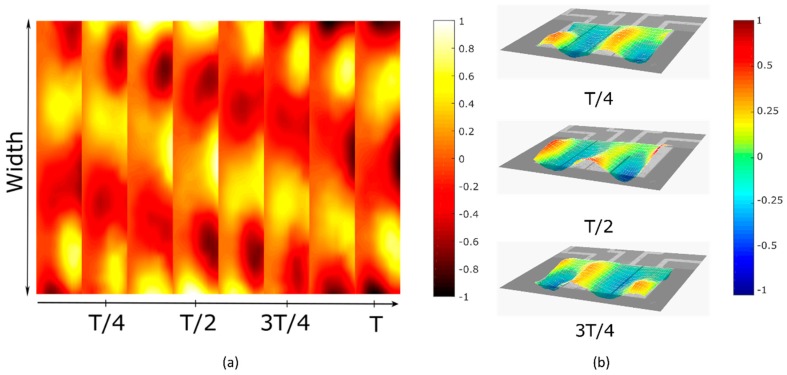
Snapshots of different times of the measured TW in isopropanol at 583.2 kHz: (**a**) 2D representation and (**b**) 3D representation (a video clip is available (Video S3)). The colors represent the normalized displacement of the cantilever. T corresponds to the period of the TW.

Generation of TWs in micromechanical plates may be applied to locomotion on surfaces. Here we demonstrate that they can also be generated in liquid, which may be an interesting actuation mechanism for underwater micro-robotics. The measurements were carried out in isopropanol. The plate and its cavity were fully immersed in the liquid and covered with a glass slider to avoid air bubbles and control the volume of liquid [21]. Modes (22) and (14) were chosen again, as they exhibited a quality factor in liquid of 47 and 120, respectively. The scheme for the actuation was similar to that used in the previous measurements in air, except that the applied voltage was increased to 7 V in liquid to improve the signal to noise ratio. Ninety phase-shifted sine waves were applied to the two outer electrodes at a frequency of 583.2 kHz. Figure 7a shows the displacement of the TW at different moments in time, and a selection of them are represented in a 3D view in Figure 7b. The animation of the movement can be seen in Video S3. It can be seen that there was an attenuation in the wave amplitude of 59% with respect to the air measurement, due to the damping induced by the liquid. The estimated phase speed was 441 m/s, and the horizontal velocity was 290 μm/s for the voltage applied. The SWR reached a value of 2.19 in 98% of the total width of the plate.

## 4. Conclusions

In summary, this paper demonstrates the feasibility of developing TWs on micro- to millimeter-scaled piezoelectrically actuated silicon-based MEMS structures, by combining two resonant modes with the proper scheme of actuation. Values of the SWR close to the ideal TW support the efficient generation mechanism. In order to validate the experimental results, FEM analysis was performed, and a good agreement between the measurements and the simulations was found. Additionally, TW generation in liquid media was presented, which revealed the potential applicability of this type of structure to locomotion in liquid media, such as miniaturized propulsion systems.

## Supplementary Materials

The following are available online at https://www.mdpi.com/2072-666X/10/5/283/s1: Video S1: 3D animation of the TW measured at 208.4 kHz in air; Video S2: 3D animation of the TW measured at 921.9 kHz in air; and Video S3: 3D animation of the TW measured at 583.2 kHz in isopropanol.
